# Expression Profiling of Non-Aflatoxigenic *Aspergillus parasiticus* Mutants Obtained by 5-Azacytosine Treatment or Serial Mycelial Transfer

**DOI:** 10.3390/toxins3080932

**Published:** 2011-08-02

**Authors:** Jeffrey R. Wilkinson, Shubha P. Kale, Deepak Bhatnagar, Jiujiang Yu, Kenneth C. Ehrlich

**Affiliations:** 1 Southern Regional Research Center, ARS/USDA, 1100 Robert E. Lee Blvd., New Orleans, LA 70124, USA; Email: jrw@juno.com (J.R.W.); deepak.bhatnagar@ars.usda.gov (D.B.); jiujiang.yu@ars.usda.gov (J.Y); 2 Department of Biology, 1 Drexel Dr., Box 85B, Xavier University of Louisiana, New Orleans, LA 70125, USA; Email: skale@xula.edu

**Keywords:** aflatoxin, microarray, 5-azacytidine, *Aspergillus parasiticus*, secondary metabolism, fluffy phenotype

## Abstract

Aflatoxins are carcinogenic secondary metabolites produced by the fungi *Aspergillus flavus* and *Aspergillus parasiticus*. Previous studies found that repeated serial mycelial transfer or treatment of *A. parasiticus* with 5-azacytidine produced colonies with a fluffy phenotype and inability to produce aflatoxins. To understand how these treatments affect expression of genes involved in aflatoxin production and development, we carried out expressed sequence tag (EST)-based microarray assays to identify genes in treated clones that are differentially expressed compared to the wild-type. Expression of 183 genes was significantly dysregulated. Of these, 38 had at least two-fold or lower expression compared to the untreated control and only two had two-fold or higher expression. The most frequent change was downregulation of genes predicted to encode membrane-bound proteins. Based on this result we hypothesize that the treatments cause changes in the structure of cellular and organelle membranes that prevent normal development and aflatoxin biosynthesis.

## 1. Introduction

Formation of the asexual reproductive structure called a conidiophore in *Aspergillus* species requires the concerted activity of a number of transcription factors and signaling proteins. Some of the transcription regulatory genes involved in this process are genes in the *Velvet* complex (*veA*, *velB*, *vosA*, and *laeA*; null mutants have a velvety appearance) and the so-called fluffy gene family (*fluG* and *flbA-E*; null mutants have a floccose or fluffy phenotype). *fluG* encodes a GSI-type glutamine synthetase that is needed for production of a diffusible factor that provides the signal for initiation of conidiophore development. *flbA* encodes a transcription factor that regulates the response to FluG. These proteins modulate the master transcription factor for conidial development, BrlA, a zinc-binding protein with two Cys_2_His_2_ domains [1,2]. In mutants of *brlA*, spores are not produced and the conidiphore stalks elongate to give a bristle appearance. Another protein, FadA is involved in G protein signaling and its inactivation is necessary for production of both sterigmatocystin, an aflatoxin precursor, and conidial development in *A. nidulans* [3]. Other genes involved in conidiophore development are *stuA* (stunted) and *medA* (medusa). These are required for proper spatial orientation of the conidophores. 

A number of studies have shown that conidiophore development and secondary metabolism are co-regulated [4,5,6,7]. In the absence of *veA*,aflatoxins (AFs), the highly toxic hydrophobic secondary metabolites produced by some *Aspergillus* species [8], are not produced. Null mutants of *laeA* also are unable to produce AFs and other secondary metabolites. LaeA is believed to act by loosening the chromatin conformation to allow transcription at secondary metabolite loci [9,10,11,12,13]. LaeA was shown to bind to VeA [9], a result providing molecular evidence that development and secondary metabolism are co-regulated. Furthermore, transcription factors NsdC and NsdD, previously only found to be required for sexual development [14,15], have now been shown to be necessary for asexual stalk formation and AF production as well.

Production of aflatoxin requires the coordinated transcription of about 30 clustered genes [16]. Two of these genes, *aflR* and *aflS* (*aflJ*), have been implicated as pathway-specific transcriptional regulators [17,18,19]. *aflR* encodes a sequence specific Cys_6_Zn_2_ DNA-binding protein, AflR, that is responsible for transcriptional activation of most, if not all, aflatoxin structural genes [20,21,22,23]. AflR activity may be regulated by LaeA [4,24]. Previous studies suggested that AflJ, a protein unique to fungi, is a co-activator of AF gene transcription and binds to AflR [18,25]. AflJ possesses three transmembrane binding helices and a microbodies *C*-terminal targeting signal and is presumed to be membrane-bound [19]. Recently, it has been shown that another level of regulation of AF formation involves the ability of *A. parasiticus* to form specialized peroxisomal vesicles necessary for the enzymatic processing of precursor metabolites after the production of the polyketide [26,27,28,29]. Coordination of aflatoxisome development with aflatoxin gene expression may be mediated by VeA and other factors. 

It was previously found that serial transfer of macerated mycelia, without allowing conidiation, produced highly stable *A. parasiticus* mutants (called sec minus strains) which had a fluffy phenotype, reduced spore pigmentation, and inability to produce AFs [7,30,31,32]. In these mutants, although expression of *aflJ* and other AF biosynthesis genes was downregulated it was possible to detect the production of some AF biosynthetic enzymes, a result suggesting that translation is occurring normally in the mutants [32]. Mutants with a “fluffy” phenotype were previously described that resulted from treatment of fungi with the cytidine analog, 5-azacytidine (5-AC) [33,34]. The 5-AC-treatment affected the formation of the conidiophore as well as conidial pigmentation. The 5-AC treatment subsequently was determined to act upon a specific target locus termed *fluF1*, a locus that still has not been molecularly defined [34]. Although 5-AC is best known as an inhibitor of DNA cytosine methyltranferases in eukaryotes, in *Aspergillus* species, where DNA methylation is lacking [33,35] or extremely low [36], 5-AC is most likely acting as a mutagen. Incorporation of 5-AC into DNA has been shown to cause C to G transversions or C to T transition mutations [37].

To provide additional insight into the causes of the phenotypic changes and the loss of aflatoxin (AF) production in fluffy mutants obtained by treatment with either 5-AC or by serial transfer we profiled the expression of genes using *A. flavus* cDNA microarrays. We found that of the 38 genes significantly downregulated two-fold or more compared to the parental strain, many were genes predicted to encode either membrane-bound proteins or transcription factors involved in regulation of developmental processes and suggest that these changes interfere with normal conidiophore development and formation of the specific organelles (aflatoxisomes [26]) required for AF formation.

## 2. Materials and Methods

### 2.1. Fungal Strains

*A. parasiticus* SRRC 143 (or SU-1); ATCC # 56775) was used as the wild-type strain. It had previously been used for numerous studies of aflatoxin biosynthesis and the entire AF gene cluster has been sequenced [16]. This strain consistently produces aflatoxins when grown on conducive media [38]. Non-aflatoxigenic *A. parasiticus* clones derived from SU-1 were prepared by serial mycelial transfer as previously described [31] or by 5-AC treatment (1 mM in A&M medium for 36 h) as described for *A. nidulans* [33]. Spores were generated on either Difco Potato-Dextrose Agar (PDA) (American Scientific Products, Charlotte, NC, USA) or 5% V8 juice, 2% agar plates.

### 2.2. Culture Conditions

**Figure 1:**
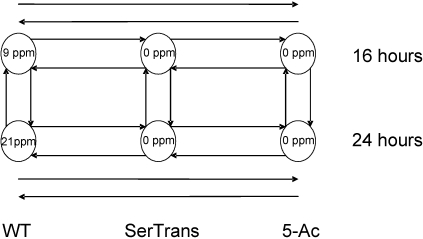
Microarray experimental design. *A. parasiticus* wild-type (WT) and clones from serial mycelial transfer (SerTrans), and 5-azacytidine-treated (5-AC) fungi were grown for 16 and 24 h on PDA medium. The average amount of aflatoxin produced across all six replicates is indicated within the circles. RNA was isolated from the mycelia at each time point and cDNAs were labeled with Cy3 (solid end of arrow) or Cy5 (arrowhead) dyes prior to microarray assay (ppm = parts per million).

For wild-type *A. parasiticus* (WT), an *A. parasiticus* 5-AC mutant, and an *A. parasiticus* serial transfer mutant (serial transfer), fresh conidia were inoculated into 200 mL A&M liquid medium (10^5^ spores/mL) and incubated at 29 °C with constant shaking (150 rpm). Mycelia were collected from clones after 16 and 24 h. Under these conditions AF production was found after 16 h (Figure 1). A total of six replicates were performed for each condition, with each replicate consisting of a total of 4 separate cultures. The four cultures were combined and 20 mL of the resultant supernatant was isolated for AF determination. The combined mycelia was flash frozen in liquid nitrogen in a pre-chilled mortar and pestle and ground to a fine powder before storage at −80 °C.

### 2.3. Aflatoxin Evaluation

AF production for all samples was evaluated by TLC and HPLC as previously described [39]. For TLC, dried samples were suspended in 50 μL of chloroform and spotted on TLC silica plates (Baker) before developing in EMW (ethanol: methanol: water; 96:3:1). Aflatoxin standard containing a mixture of B_1_, B_2_, G_1_, and G_2_ (Sigma 46300-U) was run as an external control. AF quantification was by HPLC [40].

### 2.4. Microarray Analyses

For microarray analyses, RNA was isolated from 100 mg ground mycelia by suspending in 1 mL of Puresol (Biorad). After treatment with RNase-free DNase I (Aurum Total RNA fatty and fibrous tissue kit, Biorad), the resulting RNA was converted to cDNA and labeled as previously described [41]. The EST microarrays used in this study were described previously [42,43,44]. A total of 5002 genes were arrayed at least 3 times each and 21 aflatoxin biosynthetic genes were represented separately from the EST sequences on the array. The experimental design for the microarray studies is shown in Figure 1. To minimize dye effects in separate experiments each probe was labeled with a different dye. This “dye-flip” procedure using independent microarray slides increased the reliability of the analysis and reduced dye effects. All recommendations of the minimum requirements for a microarray experiment (MIAME) checklist [45] were observed. The hybridized slides were scanned and analyzed for statistically significant different expression levels as previously described [44]. The microarray results have been deposited at the National Center for Biotechnology Information, NCBI (GEO Submissions GSE30756; tracking number 16103924).

### 2.5. Quantitative RT-PCR (qRT-PCR)

Gene specific primers (Table 1) for six genes significantly dysregulated based on the microarray results were used for a two step PCR protocol to detect transcript levels. Briefly, 750 ng of total RNA was used as the template for first-strand cDNA synthesis using oligo (dT)_20_ primers (50 °C for 50 min, cDNA synthesis System Kit (Invitrogen)) and after deactivation of the reverse transcriptase at 85 °C for 5 min, qPCR was carried out on the cDNAs using 0.5 μM each of forward and reverse primer, 2 μL of Fast Start DNA Master SYBR Green I mix (Roche Diagnostics) and 1 μL of cDNA in 20-μL capillary tubes in a Light Cycler 2.0 instrument (Roche) with the following conditions: pre-incubation (10 min at 95 °C), amplification and quantification (95° for 10 s, annealing temperature for specific primer for 8 s, 72° for 5 s), 45 cycles: melting curve (65–95° with 0.1 °C/s heating rate). Based on the standard curves abundance of transcripts for each gene was calculated by the Light Cycler software 4.0. To ensure accuracy, a non-template negative control was run for each primer pair. Generated fragments were confirmed by size separation on a 1% agarose gel. For normalization, the *A. flavus* tubulin gene (AFLA_124520) was selected. Relative expression levels were calculated as the ratio of the C_T_ for the target gene to that of the C_T_ for tubulin (Roche Applied Science Technical Note No. LC13/2001). A total of three replicates were performed for each time point. Statistical analyses were performed as previously described [44].

**Table 1:** Primers used for qRT-PCR.

NCBI or TIGR Number	Sequence (5' to 3')	Product size (bp)
*aflJ*	TGACTCTCCTTTTGCCGAATGT	250
	GGGAATGGCAACGGTGGGCG	
XM_001824706 (TC9112)	TATGAGCCCCAACGCAACAGACAG	268	
	CGGGTACGGCTCATTAGAAGGAC		
XM_002372755(TC10739)	GCCCCAGCTTACCAAACGAGA	336	
	AGGGATGAGGGGGAATGAAGTG		
XM_002373878(TC10202)	TGCGGGAAATAATACGACAGGAA	359	
	AAAGTAGCGACCCGGGCAGTAGTG		
XM_002380627(TC11938)	GACCGCATCGTGGCTTTTCTAC	334	
	AAGCATCATTCGTCATTCGTTCTG		
XM_002374352(TC9364)	CCCACCGAAGACCTGACCTACAT	322	
	CTGCCATCTGCCAAACTCCATTA		

## 3. Results

### 3.1. Loss of Aflatoxin Production after 5-Azacytosine Treatment and Serial Mycelial Transfer

The non-aflatoxigenic clones obtained after treatment with either 5-AC or repeated mycelial transfer were of several morphological types; many had a “fluffy” phenotype as previously described [30,33,46]. A total of 4 separate cultures were confirmed to have the same fluffy phenotype and loss of AF production for each of the treatments. These clones were used to prepare RNA for the microarray studies. The fluffy phenotype consisted of a dense, undifferentiated mass of aerial mycelia with only sparse conidia. While the *A. parasiticus* SU-1 wild-type cultures produced about 6.0 µg/mL AFB_1_ and 2.8 µg/mL AFG_1_ after 16 h and 14.9 and 5.8 µg/mL after 24 h, the fluffy mutant clones produced no detectable AFs or AF precursor metabolites under these conditions.

### 3.2. Microarray Transcription Profiles in 5-Azacytosine-Treated and Serial Transfer Isolated Colonies Compared to Wild-Type

In the microarray analyses approximately 20% of the genes showed a detectable signal. Microarray analyses were performed on cultures grown for 16 and 24 h in A&M medium. The ratios of mRNAs from untreated and treated cultures grown for both amounts of time were similar and only the values for the 16 h cultures are listed in Table 2 and Table 3. Of the aflatoxin cluster genes only *aflJ* was significantly downregulated more than 1.5-fold by either treatment. The microarray study detected 194 EST features that showed significant differences in expression from the wild-type (*p* ≤ 0.002). These represent about 163 unique genes. Of these genes only 35 were downregulated 1.5-fold or greater in treated cultures compared to the wild-type cultures with a high probability cut-off of 0.0001 or lower (Table 3). Most of the genes that met these criteria (Table 3) were downregulated with ratios of wild-type to treated culture ranging from 2- to 10-fold. Only two that met these criteria were upregulated. Of the downregulated genes 18 were predicted to encode proteins with membrane spanning domains typical of those in receptors involved in transport, nutrient acquisition, or signaling. Seven genes listed in Table 3 are predicted to encode transcription regulatory proteins; the remaining 14 genes fell into different categories including 7 predicted to have catalytic function. The most downregulated of the putative genes from treated clones were predicted to encode two different types of ankyrin-repeat domain proteins. Quantitative reverse-transcription (qRT) PCR was performed on mRNAs encoded by six genes (marked with a star * in Table 2 and Table 3). Correlation with the microarray results was 0.80 or higher. The genes for confirmation were chosen based on their significant downregulation compared to the parental strain. Expression of genes known to be involved in transcriptional regulation of developmental and secondary metabolite synthesis such as *veA*, *laeA*, and *nsdD* (Table 4) was not significantly affected by the treatments. A second copy of a putative LaeA-encoding methyltransferase (*laeA*-2, AFLA_035950) was slightly more than 2-fold upregulated by both the 5-AC- and serial transfer treatments. 

**Table 2:** Comparison of aflatoxin cluster gene expression in treated and untreated *A. parasiticus* SU1 detected on microarray^a^.

Gene	Ratios		Probabilities ^b^	
	Untreated *vs*. 5-AC-treated	Untreated *vs*. 5-AC-treated	Untreated *vs*. Serial transfer	Untreated *vs*. Serial transfer
*adhA*	1.22	1.10	0.4388	0.3307
aflJ^*^	1.76	1.59	0.0018	0.0015
*aflR*	1.29	1.12	0.3367	0.3465
*aflT*	1.32	1.11	0.3321	0.2423
*avfA*	1.31	1.17	0.2902	0.4271
*avnA*	1.25	1.18	0.3835	0.2162
*cypX*	1.33	1.15	0.1235	0.0835
*estA*	1.33	1.25	0.1929	0.0493
*hexA*	1.37	1.12	0.0768	0.1850
*hypB1*	1.24	0.95	0.2552	0.8827
*hypB2*	2.49	2.16	0.4722	0.5134
*moxY*	1.26	1.09	0.1828	0.1875
*nadA*	1.24	1.07	0.4882	0.4211
*nor-1*	1.41	1.12	0.0570	0.0620
*norA*	1.33	1.07	0.0553	0.2726
*omtA*	1.35	1.28	0.0422	0.0741
*omtB*	1.37	1.08	0.0385	0.2716
*ordA*	1.34	1.25	0.2363	0.1889
*ordB*	1.35	1.31	0.1566	0.0602
*pksA*	1.27	1.08	0.2264	0.1826
*sugR*	1.32	1.10	0.3869	0.3722
*vbs*	1.26	1.05	0.1547	0.1843
*ver-1*	1.23	1.10	0.7285	0.9483
*verA*	1.16	1.08	0.5312	0.8985
*verB*	1.24	1.15	0.1683	0.1181

^a ^ Genes are in the 70 kb aflatoxin biosynthetic cluster. Their functions have been previously described [16]; ^b ^ Probability determinations for microarray experiments were done as previously described [47]; * Differential expression was confirmed by qRT-PCR.

**Table 3:** Genes differentially expressed in wild-type and 5-azacytidine (5-AC)-treated and serial mycelial transfer clones^a^.

TC Number or EST ^b^	Best Match GenBank ^c^	Putative Protein	Ratios ^d^		Probabilities ^d^	
			Untreated *vs.* 5-AC-treated	Untreated *vs.* Serial transfer	Untreated *vs.* 5-AC-treated	Untreated *vs.* Serial transfer
TC8344	XM_001824592	HET&ankyrin domain protein	10.02	8.53	0.0000	0.0000
TC10739 *	XM_002372755	Ankyrin repeat-containing protein	5.69	4.52	0.0000	0.0000
TC9364 *	XM_002374352	FG-GAP (integrin) repeat protein	4.79	3.97	0.0000	0.0000
TC9256	XM_002375859	Nitrite reductase (NiiA)	4.58	3.38	0.0000	0.0000
NAGAO52TV	XM_002380681	Polysaccharide synthase Cps1	4.54	4.72	0.0000	0.0000
TC10370	XM_002384463	Opsin	4.18	3.49	0.0000	0.0000
TC8557	XM_001827098	alpha1,3-glucan synthase	3.71	2.62	0.0000	0.0000
TC10767 *	XM_003096248	Integral membrane protein	3.54	2.49	0.0000	0.0002
TC10268	XM_002383371	12 kda heat shock protein	3.53	3.48	0.0000	0.0000
TC9112 *	XM_001824706	GPI-anchored protein	3.45	3.00	0.0000	0.0000
TC10523	XM_002374369	Integral membrane prot-exocytic transport from Golgi	3.40	3.53	0.0000	0.0000
NAGCY20TV	XM_002379925	Gal4 transcription factor-AFLA_139560	3.28	3.20	0.0000	0.0000
NAGAE41TV	XM_002382445	Calcium binding protein Caleosin	3.23	3.09	0.0000	0.0000
TC10524	XM_002374369	Exocytic transport protein	3.21	3.28	0.0000	0.0000
TC10334	XM_002379834	GABA-permease	3.11	2.69	0.0000	0.0000
TC11140	XM_002383723	MFS-transporter-related	3.02	2.18	0.0000	0.0007
TC11938 *	XM_002380627	EsdC	2.98	2.71	0.0000	0.0000
NAFCO41TV	XM_001819214	Transcriptional regulatory protein pro1 (HLH protein)	2.88	2.35	0.0000	0.0000
TC11262	XM_002384600	Helix-loop-helix DNA-binding domain protein	2.75	2.40	0.0000	0.0001
TC10225	XM_003189329	SH3 domain kinase binding protein (predicted membrane protein)	2.70	2.47	0.0000	0.0000
TC8802	XM_002378642	Ser-thr rich protein AOS28(solid-state culture specific gene)	2.66	2.63	0.0000	0.0000
TC11294	XM_002384659	Arrestin domain protein	2.54	1.92	0.0000	0.0007
TC8781	XM_002375350	MAPEG superfamily protein pfam01124	2.45	1.81	0.0000	0.0003
TC10523	XM_002374369	Hypothetical protein	2.40	2.54	0.0000	0.0000
TC10950	XM_002377280	RTA domain protein (RTA1 superfamily, pfam04479)	2.15	1.82	0.0000	0.0000
TC8911	XM_002383317	Aspartic endopeptidase; aspartyl proteinase	2.13	1.82	0.0000	0.0002
TC8680	XM_002382580	GPI-anchored glycoprotein (AFLA_004200)	2.03	1.98	0.0000	0.0000
TC9120	XM_002381180	bZIP transcription factor ATFB (atf21, AFLA_094010)	2.02	1.96	0.0000	0.0000
TC8511	XM_002383753	Hypothetical protein	1.92	2.11	0.0000	0.0000
NAFBR39TV	XM_002378094	alpha/beta hydrolase domain protein	1.90	1.66	0.0000	0.0002
TC9105	XM_001824752	Hypothetical protein	1.87	1.96	0.0000	0.0000
TC10397	XM_002379378	PHD finger and SET domain protein	1.67	1.75	0.0000	0.0000
TC9269	XM_002384586	Metallophophodiesterase (AFLA_119200)	1.66	1.55	0.0000	0.0001
TC9718	XM_002372994	CVNH superfamily (cyanovirin-N family, pfam08881)	0.53	0.61	0.0000	0.0001
NAFEY64TV	XM_002377133	Asparagine synthase (glutamine-hydrolyzing)	0.49	0.52	0.0001	0.0003

^a ^ Only genes expressed at the 99.9% confidence level with ratio of 1.5-fold or higher or 0.5 or lower are shown. A star (*) indicates that the difference was confirmed by qRT-PCR; ^b ^ DNA sequence labels correspond to expressed sequence tags (EST) or tentative consensus sequences (TC) as per the DFCI *A. flavus* Gene Index (http://weir.statgen.ncsu.edu/cgi-bin/gbrowse/aflavus_dec_2005/#search); ^c ^ Matches to GenBank sequences were based on Blast of the EST sequence against the GenBank Nucleotide collection (http://blast.ncbi.nlm.nih.gov/Blast.cgi?PROGRAM=blastn&BLAST_PROGRAMS=megaBlast&PAGE_TYPE=BlastSearch&SHOW_DEFAULTS= on&BLAST_); ^d ^ Ratios and probabilities are for data from the 16 h time point. Substantially similar data were obtained at 24 h.

**Table 4:** Comparison of expression of some known genes involved in secondary metabolism, and fungal development.

Gene	Ratios		Probabilities			Expressed Sequence Tag (EST)	GenBank Accession Number	AFLA #
	Untreated *vs.* 5-AC-treated	Untreated *vs*. Serial transfer		Untreated *vs.* 5-AC-treated	Untreated *vs.* Serial transfer			
*laeA*	0.75	0.77		0.8998	0.8874	Unknown	XM_002374798	033290
*laeA-1*^1^	0.41	0.40		0.0002	0.0002	TC9243	XM_002375064	035950
*veA*	0.76	0.68		0.0307	0.0041	TC11465	XM_002380164	066460
*nsdD*	1.83	0.99		0.0145	0.9512	TC10891	XM_002376000	020210A

^1^ Another gene encoding a LaeA-like protein (AFLA_121330) is found in the fungal genome but was not annotated on the EST microarray.

## 4. Discussion

The morphologically variant *Aspergillus parasiticus* strains obtained by either 5-AC treatment or serial mycelial transfer showed strikingly similar expression profiles to *A. flavus* clones created by a slightly different serial transfer method [44]. The most dysregulated genes in clones obtained by both serial mycelial transfer and 5-AC treated mutants are predicted to encode membrane-bound proteins which could act as receptors or transporters or be involved in signaling or signal transduction. Of these, the most dysregulated gene (XM_001824592) encodes a protein predicted to have both protein kinase, HET and ankyrin repeat domains [48]. Such proteins typically are involved in protein-protein interactions. An ankyrin repeat is a 33-aa motif in proteins consisting of two alpha helices separated by loops and present in proteins known to be involved in signaling, transcription initiation, cell cycle regulation, ion transport, and signal transduction [49]. Therefore dysregulation of expression of this type of protein could be expected to cause pleiotropic effects on cell morphology and development. The putative HET functionality is found in proteins that mediate heterokaryon incompatibility reactions. Such functionality could play a role in mediating the activity of the FluG factor that induces conidial development and secondary metabolism and in this case, may be critical for normal development and AF production.

Another gene (XM_002384463) significantly downregulated by either treatment is predicted to encode opsin, a protein with transmembrane helices that may bind a photoreactive chromophore [50]. Opsins are bound to the lipid bilayer, and when involved in light signaling, undergo a light-induced conformational change necessary to activate a G protein signaling cascade. Also represented on the microarray as significantly downregulated is an FG-Gap repeat-containing protein. FG-GAP repeats are found in proteins near the *N*-terminus of integrin alpha chains, a region that has been shown to be important for ligand binding in membranes. Integrins act as receptors and can mediate attachments between cells or mediate interactions with the environment. They also play a role in cell signaling and define cellular shape, mobility, and regulate the cell cycle. Integrins can mediate outside-in as well as inside-out signaling [51].

Another intriguing hit among the most dysregulated genes is one (XM_002382445) predicted to encode the calcium-binding protein, caleosin [52]. Caleosins contain calcium-binding domains and have an oleosin-like association with lipid bodies. Caleosins are present at relatively low levels in plants and are mainly bound to microsomal membrane fractions at the early stages of seed development. As the seeds mature, overall levels of caleosins increase dramatically and are associated almost exclusively with lipid storage bodies. A similar function for these proteins in fungi could be an association with the specialized peroxisomal structures necessary for biosynthesis of the hydrophobic aflatoxin intermediates (see below). 

**Figure 2:**
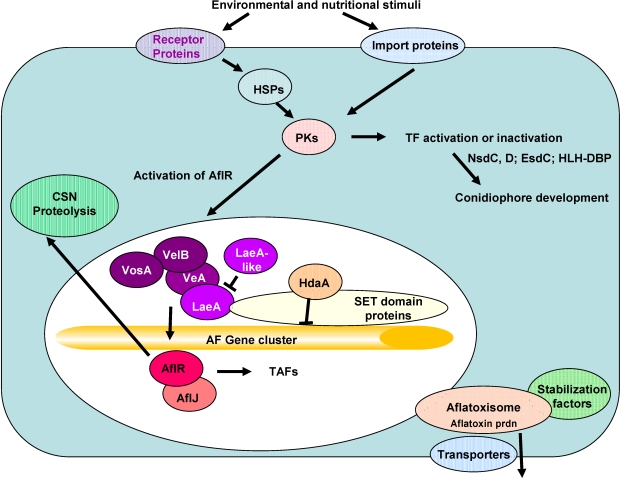
Model to explain how the dysregulated genes in the microarray study might affect aflatoxin production and conidiophore development. Abbreviations: HSPs, heat shock proteins; PKs, protein kinases; CSN, COP9 signalosome involved in protein degradation or prevention from degradation; TF, transcription factor; HLH-DBP, helix-loop-helix DNA-binding protein; TAFs, transcription-activating factors.

Other genes predicted to encode membrane-bound proteins are XM_001824706 (a GPI-anchored protein), XM_002374369 (a protein involved in exocytic transport), XM_002379834 (a GABA permease), XM_002383723 (a MFS transporter) and XM_003189329 (an SH3 domain kinase binding protein). All of these proteins could affect nutrient transport or transmission of environmental signals [53,54]. GPI (glycosylphosphatidylinositol)-anchored proteins contain a signal peptide that directs them to the endoplasmic reticulum, after which, the hydrophobic *C*-terminus is removed and replaced by the GPI-anchor. The protein is subsequently transferred to the Golgi apparatus and finally to the extracellular space. Since the anchor is the sole means of attachment of such proteins to the membrane, cleavage of the group by phospholipases results in controlled release of the protein from the membrane. Such proteins could mediate exocytic transport of the hydrophobic aflatoxins or other intermediates within and outside the fungal cell [55]. Hypothetical roles of these proteins are consistent with the changes in development and secondary metabolism found in the mutants created by the serial transfer or 5-AC treatments. Their involvement in the developmental changes in clones selected by the treatments remains to be proven. Downregulation of expression of genes encoding membrane-bound proteins described above could explain the loss of normal development of asexual structures required for proper formation of conidia. How this developmental change affects AF production is not clear. Further work is needed to determine if deletions of some of these genes produce clones with the same phenotype obtained by serial mycelial transfer or treatment with 5-AC. It is possible that more than one gene alteration is necessary for creating the non-aflatoxigenic mutants profiled in our study. Putative roles for the genes are shown schematically in Figure 2.

Other significantly dysregulated genes listed in Table 3 are genes predicted to encode transcription control proteins. These include a protein involved in regulation of early sexual development (XM_002380627, EsdC), a Gal4-type transcription factor, AFLA_139560, a helix-loop-helix domain DNA-binding protein (XM_002384600), an ATF/CREB family bZIP-type transcription factor (XM_002381180) that may bind to the cAMP-responsive element (CRE) to stimulate CRE-dependent transcription [56], and a PHD finger and SET domain protein (XM_002379378) [57,58,59] that could bind to histone H3 tri-methylated on lysine‑4 (H3K4me3) or H3 tri-methylated lysine‑9 (H3K9me3). SET domain proteins are mediator proteins that activate or repress chromatin activity depending on the presence of other chromatin proteins. Dysregulation of the Gal4 transcription factor, AFLA_139560 is particularly intriguing because it is located on chromosome 3 quite close to the cyclopiazonic acid (CPA) and adjoining aflatoxin gene clusters. 

Since the serial transfer or 5-AC created mutants examined by our expression profiling studies showed almost normal expression of most AF biosynthetic genes (Table 2), it is possible that the lack of aflatoxin production results from an inability to form the proper organelles necessary for AF biosynthesis. Recent work showed that a specialized peroxisomal vesicle is needed for proper positioning of the biosynthetic enzymes [26,27,28,29]. Such vesicles, termed aflatoxisomes, are specialized for AF biosynthesis and may allow the coordination of catalytic steps needed for the proper tethering and ultimately export of the developing hydrophobic AF metabolite. If such a structure is not able to be formed properly in the mutant cells, the biosynthetic proteins cannot catalyze the conversions needed for AF formation. Loss of this structure due to downregulation of genes that are involved in membrane function and/or conidiophore development is consistent with the fluffy phenotype and non-aflatoxigenicity common to both the 5-AC and serial mycelial transfer mutants. If this is true, a level of control of AF production beyond transcription and translation may exist that could provide a target for remediation of AF formation in susceptible crops. 

Expression of *laeA* (AFLA_033290), the transcriptional activator of secondary metabolite biosynthesis and development-specific transcription activators, was not found to be affected in clones obtained by either treatment. However, expression of a gene encoding an *laeA* homolog (AFLA_035950) was significantly upregulated in clones obtained by both treatments. This protein is similar to an LaeA antagonist found in *A. nidulans *(LaeA-1) that, based on previous studies [60] (N. Keller, personal communication) may interfere with the activity of LaeA. If LaeA-1 interferes with LaeA’s ability to regulate aflatoxin cluster gene expression it must do so without significantly altering expression of most of the AF biosynthesis genes. 

## 5. Conclusions

In *A. parasiticus* both 5-AC treatment and serial mycelial transfer lead to loss of AF production and abnormal conidophore development. The microarray experiments reveal that similar genes are dysregulated by the two treatments. Of the 35 most dysregulated genes, more than half are predicted to be involved in membrane functions such as signaling, transport or guiding protein interactions. Downregulation of these types of genes could account for the phenotypic changes in the mutant cultures. In our study, loss of the ability to produce aflatoxins by these mutants is not a result of transcription inhibition of aflatoxin biosynthesis genes or translation of the mRNA. We hypothesize that the most likely explanation for the non-aflatoxigenicity of the treated cultures is their inability to form the proper vesicle structure for coordinated enzymatic conversion of AF precursors to stable metabolites. 
